# Prospective Study to Evaluate the Role of Multidetector Computed Tomography in Evaluation of Paranasal Sinus Pathologies

**DOI:** 10.7759/cureus.24011

**Published:** 2022-04-10

**Authors:** Tarim Usmani, Eram Fatima, Vivek Raj, Kashish Aggarwal

**Affiliations:** 1 Radiodiagnosis, Career Institute of Medical Sciences and Hospital, Lucknow, IND

**Keywords:** sinusitis, polyp, paranasal sinus, fess, mdct

## Abstract

Background: The use of computed tomography (CT) combined with functional endoscopic sinus surgery (FESS) has empowered the modern sinus surgeon to treat patients more effectively, facilitating reduced morbidity and complications. This study aimed to evaluate the role of multidetector computed tomography (MDCT) in the evaluation of paranasal sinus pathologies.

Materials & Methods: This cross-sectional study was conducted among the adult subjects attending the department of radiology at a tertiary care center in Lucknow for CT scan of paranasal sinuses for suspected paranasal sinus pathology from August 2018 to January 2020. The study included subjects above 12 years of age visiting the facility for CT of paranasal sinuses with a suspected paranasal sinus pathology and were also undergoing FESS.

Results: A total of 74 patients falling in the sampling frame were enrolled in the study. Majority of cases were aged <40 years (n=41; 55.4%). Age group of 21-30 years was most affected (24.3%). Mean age of patients was 38.39±14.48 years. Inflammatory pathologies diagnosed on MDCT-included sinusitis (n=25), sinonasal polyps (n=17), sinusitis with polyps (n=15), and mucocele (n=2), respectively. FESS/histopathological diagnosis was done in 73 cases and revealed inflammatory pathology in 59/73 (80.8%) and neoplastic pathology in 14 (19.2%) cases. FESS/histopathological break up of inflammatory pathologies included 22 cases of sinusitis, 20 cases of sinonasal polyps, 14 cases of sinusitis with polyps, and three cases of mucocele. Agreement between MDCT and final diagnosis was seen in 67/74 (90.5%) cases assessed.

Conclusion: The findings of the present study showed that MDCT is a useful modality for preoperative assessment of paranasal sinuses. With its high precision in diagnosis, it can help in further treatment planning and management in patients with paranasal sinus pathologies.

## Introduction

The nasal cavity, which extends from the external nares to the nasopharynx, is lined by ciliated nasal mucosa and is surrounded by a collection of air-filled cavities called the paranasal sinuses (PNS). Because they are pneumatic diverticula from the primitive nasal cavity, the mucosa lining of PNS is identical to that of the nasal cavity, therefore, any pathological alterations affecting the nasal mucosa might transfer to the PNS [1-3].

The combined use of computed tomography (CT) with functional endoscopic sinus surgery (FESS) has led to more effective treatment of patients by the modern sinus surgeon, thereby facilitating reduced morbidity and complications. To be able to successfully treat patients with paranasal sinus pathology, physicians must be capable of reading and interpreting sinus CT scans. Knowledge of the anatomy of PNS and their variant features forms the groundwork from which radiologic interpretation begins. The readers can further augment their ability to understand sinus CT findings by familiarizing themselves with the cross-sectional anatomy and radiologic landmarks on patients’ CT scans, along with respective clinical correlations. Recent evidence has shown that multidetector computed tomography (MDCT) provides a better and panoramic view of the paranasal sinus and helps to visualize the varying pathologies with considerable clarity [4-6].

Given the advancement of MDCT in giving an accurate diagnosis of paranasal sinus diseases, the current study is being suggested to examine the role of MDCT in diagnosing paranasal sinus pathologies and to connect it with the findings of FESS. The present study aimed to evaluate the role of multidetector CT in the evaluation of paranasal sinus pathologies.

## Materials and methods

This cross-sectional study was conducted among the adult subjects attending the department of radiology at a tertiary care center in Lucknow for CT scan of PNS for suspected paranasal sinus pathology from August 2018 to January 2020. The study included subjects above 12 years of age visiting the facility for CT of PNS with a suspected paranasal sinus pathology and undergoing FESS. Individuals who did not wish to undergo FESS or were unwilling to participate in the study were excluded from the study. The study was approved by the Career Institute of Medical Sciences Ethics Committee with approval number CIMSH8787.

After obtaining informed consent and demographic information, the duration and nature of complaints were noted. All the patients were then subjected to MDCT evaluation. The parameters which were focused upon during MDCT evaluation were for septal deviation, concha bullosa of middle and inferior turbinates, paradoxical concha, changes of uncinate process, Haller cell, sphenoid sinus variations, hypoplasia of frontal sinus, and maxillary sinus. The findings were validated during FESS. Histopathology, where necessary and indicated, was performed.

## Results

A total of 74 patients falling in the sampling frame were enrolled in the study. Majority of cases were aged <40 years (n=41; 55.4%). Age group of 21-30 years was most affected (24.3%). Mean age of patients was 38.39±14.48 years (Table 1). Majority of patients were females (n=41; 55.4%) (Table 2). Male to female ratio of the study was 0.8. Headache was the most common presenting complaint (n=57; 77%) followed by altered smell (n=37; 50%), nasal obstruction (n=28; 37.8%), nasal discharge (n=27; 36.5%), facial puffiness (n=18; 24.3%), nasal mass (n=13; 17.6%), and nasal bleed (n=11; 14.9%) (Table 3). Maxillary sinus was most involved (n=41; 55.4%) followed by frontal (n=19; 25.7%), ethmoid (n=13; 17.6%), and sphenoid sinus (n=11; 14.9%), respectively (Table 4).

**Table 1:** Distribution of patients according to age. SD: standard deviation Mean age ± SD (range) in years = 38.39 ± 14.48 (14-67)

Age group	Number of patients	Percentage
<20 Years	10	13.5
21–30 Years	18	24.3
31–40 Years	13	17.6
41–50 Years	15	20.3
51–60 Years	12	16.2
61–70 Years	6	8.1

**Table 2:** Distribution of patients according to gender.

Sex	Number of patients	Percentage
Male	33	44.6
Female	41	55.4

**Table 3:** Distribution of patients according to presenting complaints.

Presenting complaints	Number of patients	Percentage
Nasal obstruction	28	37.8
Nasal discharge	27	36.5
Headache	57	77.0
Facial puffiness	18	24.3
Altered smell	37	50.0
Nasal mass	13	17.6
Nasal bleed	11	14.9

**Table 4:** Distribution of patients according to the region involved.

Region involved	Number of patients	Percentage
Maxillary	41	55.4
Frontal	19	25.7
Ethmoid	13	17.6
Sphenoid	11	14.9

On MDCT, maximum number of cases were diagnosed as inflammatory disorders (n=59; 79.7%) followed by neoplastic lesions (n=14; 18.9%) that included six (8.1%) malignant and eight (10.8%) benign pathologies. There was one (1.4%) case with anatomical variation who was diagnosed to have deviated nasal septum resulting in obstruction. Among different inflammatory pathologies-sinusitis (n=25) was the most common pathology followed by sinonasal polyps (n=17), sinusitis with polyps (n=15), and mucocele (n=2), respectively (Table 5).

**Table 5:** Various findings on MDCT. MDCT: multidetector computed tomography; DNS: deviated nasal septum

MDCT evaluation findings	Number of patients	Percentage
Inflammatory	59	79.7
Sinusitis	25	33.8
Sinonasal polyps	17	23.0
Sinusitis with polyps	15	20.3
Mucocele	2	2.7
Neoplastic lesions	14	18.9
Malignant	6	8.1
Benign	8	10.8
Anatomical variation		
Deviated nasal septum (DNS)	1	1.4

FESS/histopathological diagnosis was done in 73 cases only as one case detected with deviated nasal septum did not participate in subsequent evaluation. Inflammatory pathology was diagnosed in 59/73 (80.8%) cases and included 22 cases of sinusitis, 20 cases of sinonasal polyps, 14 cases of sinusitis with polyps, and three cases of mucocele. A total of 14 (19.2%) cases were diagnosed as neoplastic lesions and included five malignant and nine benign cases (Table 6).

**Table 6:** Various findings on FESS/histopathology. FESS: functional endoscopic sinus surgery

FESS/Histopathological diagnosis	Number of patients	Percentage
Inflammatory	59	80.8
Sinusitis	22	30.1
Sinonasal polyps	20	27.4
Sinusitis with polyps	14	19.2
Mucocele	3	2.7
Neoplastic lesions	14	19.2
Malignant	5	6.8
Benign	9	12.2

On histopathology, among different benign pathologies (n=9), nasopharyngeal angiofibroma (n=3; 33.3%) was most common followed by fibrous dysplasia and inverted papilloma (n=2; 22.2% each). There was one (11.1%) case each of neurofibroma and squamous papilloma. Among the malignant pathologies (n=5), all five (100%) cases were diagnosed as squamous cell carcinoma (Table 7).

**Table 7:** Various histopathological findings.

Histopathological diagnosis	Number of patients	Percentage
*Benign (n=9)*		
Nasopharyngeal angiofibroma	3	33.3
Fibrous dysplasia	2	22.2
Inverted papilloma	2	22.2
Neurofibroma	1	11.1
Squamous papilloma	1	11.1
Malignant (n=5)		
Squamous cell carcinoma	5	100

For neoplastic lesions and deviated nasal septum, MDCT had an absolute accuracy (100% sensitive and 100% specific). Among other pathologies, for sinusitis MDCT was 100% sensitive and 94.1% specific, for polyps it was 91.2% sensitive and 97.4% specific, for sinonasal polyps, it was 85% sensitive and 100% specific, for sinusitis with polyps it was 100% sensitive and 98.3% specific, for mucocele it was 66.7% sensitive and 100% specific, for malignancy it was 80% sensitive and 97.1% specific and for benign lesions, it was 77.8% sensitive and 98.4% specific. The positive predictive value of MDCT ranged from 66.7% (malignancy) to 100% (sinonasal polyps, neoplastic lesions, and DNS). The negative predictive value of MDCT ranged from 92.7% (polyps) to 100% (neoplastic lesions, DNS, sinusitis, and sinusitis with polyps). Accuracy of MDCT ranged from 94.5% (polyps) to 100% (neoplastic lesions and DNS) (Table 8).

**Table 8:** Diagnostic efficacy of MDCT against FESS/HPE. FESS: functional endoscopic sinus surgery; HPE: histopathological examination; MDCT: multidetector computed tomography; TP: true positive; FP: false positive; FN: false negative; TP: true positive; PPV: positive predictive value; NPV: negative predictive value; DNS: deviated nasal septum

FESS/HPE	MDCT diagnosis				MDCT diagnostic efficacy				
	TP	FP	FN	TP	Sensitivity	Specificity	PPV	NPV	Accuracy
Neoplastic lesions	14	0	0	60	100	100	100	100	100
DNS	1	0	0	73	100	100	100	100	100
Sinusitis	22	3	0	48	100	94.1	88.0	100	95.9
Polyps	31	1	3	38	91.2	97.4	96.9	92.7	94.5
Sinonasal polyps	17	0	3	53	85.0	100	100	94.6	95.9
Sinusitis with polyps	14	1	0	58	100	98.3	93.3	100	98.6
Mucocele	2	0	1	70	66.7	100	100	98.6	98.6
Malignant	4	2	1	66	80.0	97.1	66.7	98.5	95.9
Benign	7	1	2	63	77.8	98.4	87.5	96.9	95.9

## Discussion

The clinical profile of paranasal sinus pathologies is quite generalized, and diagnosis based on clinical profile alone is often misleading. However, the complex anatomy of the PNS is difficult to be illustrated on normal radiological imaging. In recent years, the emergence of newer imaging modalities has extended a new dimension to the diagnosis of paranasal sinus pathologies. The investigation of sinonasal pathology and anatomic variants using computerized tomography (CT) has proven indispensable in preoperative planning for FESS [7]. Assessment of chronic rhinosinusitis by CT scan is a reliable test. The CT findings in patients with paranasal sinus pathologies remain consistent over time [8].

The present study showed a dominance of features like headache (77%), alteration in smell (50%), nasal obstruction (37.8%), and nasal discharge (36.5%) as the most common presenting complaints. Although the spectrum of presenting complaints remains the same in different studies, however, there are proportional differences in the type of dominant complaints in different studies. Verma et al. [9] in their study reported nasal obstruction (82%), nasal discharge (66%), headache, and allergic symptoms (52%) as the dominant presenting complaints, however, Kandukuri and Phatak [10] reported nasal obstruction and discharge as the most common finding.

The present study showed involvement of maxillary sinus to be the most common (n=41; 55.4%) followed by frontal (n=19; 25.7%), ethmoid (n=13; 17.6%), and sphenoid sinus (n=11; 14.9%), respectively. The profile of involved site in different case series has shown variability, however, maxillary sinus remains to be the most commonly affected region in most of the series. In their study, Kushwah et al. [11] found maxillary sinus to be involved in 88% of cases followed by sphenoidal (14%) and ethmoidal (8%) regions. Bagul (2016) [12] in their study found involvement of maxillary, ethmoidal, frontal, and sphenoidal regions in 86%, 54%, 31%, and 21% cases, respectively, thus showing involvement of multiple regions in many cases. PNS, owing to their proximity and continuity, are often affected simultaneously. In the present study, too, multiple regions were involved in several cases.

In their study, Kanwar et al. [13] reported the final diagnosis to be non-specific inflammation suggestive of sinusitis, inflammatory polyps, antrochoanal polyp, fungal sinusitis, and poorly differentiated carcinoma in 57.1%, 24.1%, 7.6%, 6.5%, and 4.3% cases, respectively. Bagul [12] identified inflammatory disease, sinusitis, polyposis, neoplastic, miscellaneous, and other pathologies in 66 (60%), 40 (36.4%), 18 (2.7%), 36 (32.7%), eight (7.3%), and seven (6.4%) patients, respectively. Kushwah et al. [11] in their study reported non-specific inflammatory diseases as poorly differentiated carcinoma, inflammatory polyp, angiofibroma, inverted papilloma, fungal sinusitis, and mucocele in 64%, 14%, 8%, 6%, 4%, 2%, and 2% cases, respectively. Chaitanya and Raviteja [14] in their study on final diagnosis found 51.9% cases as chronic sinusitis, 30.8% as polyp, 7.7% as fungal sinusitis, and 9.6% as other pathologies.

On evaluating the profile of neoplastic lesions, among nine benign cases, nasopharyngeal angiofibroma (n=3; 33.3%) was most common followed by fibrous dysplasia and inverted papilloma (n=2; 22.2% each). There was one (11.1%) case each of neurofibroma and squamous papilloma while all the five (100%) cases of malignancy were squamous cell carcinoma. In a study dedicated to evaluation of neoplastic lesions of PNS only, Sivalingam et al. [15] reported angiofibroma (41.6%), papilloma (29%), and osteoma (8%) as the major benign, and squamous cell carcinoma (43%), adenocarcinoma (12%), and lymphoma (25%) as the major malignant pathologies. Kushwah et al. [11] in their study had three out of total five cases with benign pathologies to be angiofibroma and the remaining two to be inverted papilloma. In their study, they reported all the seven cases with malignancy as poorly differentiated carcinoma and did not exactly report the histopathological type. Verma et al. [9] on the other hand, in their study, reported one case of adenocarcinoma and one case of squamous cell carcinoma. In fact, owing to low rate of neoplastic lesions in different studies, there are incidental differences in different histopathological outcomes.

In the present study, an agreement between final diagnosis and diagnosis by MDCT was observed in 67/74 (90.5%) cases. Kushwah et al. [11] in their study found this agreement to be slightly higher at 94%. Chaitanya and Raviteja [14] also reported an agreement of 95.2% between CT and endoscopy findings. However, Rao [16] in her study reported an agreement of 88% between CT and endoscopic findings. In the present study, the cases that missed diagnosis included four cases of sinonasal polyps diagnosed as sinusitis on MDCT, two cases of benign lesions diagnosed as a malignancy on MDCT, and one case each of mucocele and malignancy diagnosed as sinusitis with polyps and benign lesion, respectively, on MDCT.

The study found MDCT to be 100% sensitive and 94.1% specific for sinusitis, 91.2% sensitive and 97.4% specific for polyps, 85% sensitive and 100% specific for sinonasal polyps, 100% sensitive and 98.3% specific for sinusitis with polyps, 66.7% sensitive and 100% specific for mucocele, 80% sensitive and 97.1% specific for malignancy, and 77.8% sensitive and 98.4% specific for benign cases.

Different studies have reported high sensitivity and specificity of MDCT for various PNS pathologies. Kushwah et al. [11] in their study reported that CT had a sensitivity and specificity of 100% and 93.8% for chronic cervicitis, 100% and 93.8% for polyp, and 93.8% and 100% for other pathologies. Rao [16] reported that CT had a sensitivity and specificity of 86% and 96.5% for chronic sinusitis, 96.15% and 95.83% for polyps, 71.4% and 93.02% for fungal sinusitis, and 100% for other pathologies.

MDCT evaluation of PNS, as seen in the present study, was found to be quite useful in ascertaining the underlying pathology. The findings of the study matched with the contemporary literature. In view of the useful information provided by MDCT, we recommend its use as an essential preoperative/diagnostic assessment among patients scheduled to undergo FESS.

## Conclusions

The findings of the present study showed that MDCT is a useful modality for preoperative assessment of paranasal sinus, with different studies reporting high sensitivity and specificity for various PNS pathologies. With its high precision in diagnosis, it can help in further treatment planning and management in patients with paranasal sinus pathologies. Its use has proven indispensable in preoperative planning for FESS. This combined approach has led to more effective treatment of patients by the modern sinus surgeon, thereby facilitating reduced morbidity and complications.
